# Hepatocellular carcinoma diagnosis using a novel electrochemiluminescence immunoassay targeting serum IgM-free AIM

**DOI:** 10.1007/s12328-021-01567-4

**Published:** 2022-01-04

**Authors:** Tomo Shimizu, Takashi Sawada, Tomohide Asai, Yuka Kanetsuki, Jiro Hirota, Michihisa Moriguchi, Tomoaki Nakajima, Toru Miyazaki, Takeshi Okanoue

**Affiliations:** 1 Tsukuba Research Institute Research and Development Division, Sekisui Medical Co., Ltd., 3-3-1, Koyodai, Ryugasaki, Ibaraki 301-0852 Japan; 2 Department of Molecular Gastroenterology and Hepatology, Graduate School of Medical Science, Kyoto Prefectural University of Medicine, Kyoto, 602-8566 Japan; 3 Department of Hepatology, Sapporo Kosei General Hospital, Hokkaido, 060-0033 Japan; 4 Laboratory of Molecular Biomedicine for Pathogenesis, Center for Disease Biology and Integrative Medicine, Faculty of Medicine, The University of Tokyo, Tokyo, 113-0033 Japan; 5 LEAP, Japan Agency for Medical Research and Development, Tokyo, 113-0033 Japan; 6 Laboratoire d’ImmunoRhumatologie Moléculaire, Plateforme GENOMAX, Institut National de la Santé et de la Recherche Médicale UMR_S 1109, Faculté de Médecine, Fédération Hospitalo-Universitaire OMICARE, Fédération de Médecine Translationnelle de Strasbourg, Laboratory of Excellence TRANSPLANTEX, Université de Strasbourg, Strasbourg, France; 7 The Institute for AIM Medicine, Tokyo, 101-0047 Japan; 8 Department of Gastroenterology and Hepatology, Saiseikai Suita Hospital, Osaka, 564-0013 Japan

**Keywords:** Non-alcoholic steatohepatitis, Hepatocellular carcinoma, IgM-free apoptosis inhibitor of macrophage, Electrochemiluminescence immunoassay, Diagnosis

## Abstract

**Background:**

Recent increases in the number of patients with non-alcoholic steatohepatitis (NASH) warrant the identification of biomarkers for early detection of hepatocellular carcinoma (HCC) associated with NASH (NASH-HCC). IgM-free apoptosis inhibitor of macrophage (AIM), which generally associates with IgM in blood and exerts its biological function by dissociation from IgM, may serve as an effective biomarker for NASH-HCC. Here, we established a fully automatic and high-throughput electrochemiluminescence immunoassay (ECLIA) to measure IgM-free AIM and investigated its efficacy in diagnosing NASH-HCC and viral HCC.

**Methods:**

IgM-free AIM levels were measured in 212 serum samples from patients with, or without, HCC related to NASH, hepatitis B virus, and hepatitis C virus, using ECLIA. We also developed an ECLIA for measuring both IgM-free and IgM-bound AIM and investigated the existing form of AIM in blood by size-exclusion chromatography.

**Results:**

IgM-free AIM levels were significantly higher in the HCC group than in the non-HCC group, regardless of the associated pathogenesis. Moreover, the area under the receiver operating curve for IgM-free AIM was greater than that for conventional HCC biomarkers, alpha-fetoprotein or des-γ-carboxy prothrombin, regardless of the cancer stage. ECLIA counts of IgM-free AIM derived from samples fractionated by size-exclusion chromatography were significantly higher in patients with NASH-HCC than in healthy volunteers and in patients with non-alcoholic fatty liver and NASH.

**Conclusions:**

Serum IgM-free AIM may represent a universal HCC diagnostic marker superior to alpha-fetoprotein or des-γ-carboxy prothrombin. Our newly established ECLIA could contribute to further clinical studies on AIM and in vitro HCC diagnosis.

**Supplementary Information:**

The online version contains supplementary material available at 10.1007/s12328-021-01567-4.

## Introduction

Non-alcoholic fatty liver disease (NAFLD) is the most common chronic liver disease in many countries and is closely associated with metabolic syndrome [1–4]. NAFLD is classified as non-alcoholic fatty liver (NAFL) (benign form) and non-alcoholic steatohepatitis (NASH) (active form), which could progress to cirrhosis and hepatocellular carcinoma (HCC) [5]. Most HCCs are caused by viral hepatitis; however, therapeutic advances have contributed to viral clearance in many patients with hepatitis C, while those with hepatitis B are often clinically controlled using nucleotide/nucleoside analogs [6, 7]. Consequently, the annual incidence of hepatitis virus-induced HCC has gradually decreased. In contrast, the number of patients with obesity and diabetes has increased; as such, NASH-HCC incidence has increased against the backdrop of metabolic syndrome NASH associated with obesity, diabetes, dyslipidemia, and hypertension, necessitating the development of appropriate countermeasures that can be applied worldwide [8].

Alpha-fetoprotein (AFP) and des-γ-carboxy prothrombin (DCP) are widely recognized HCC biomarkers. For early HCC detection, surveillance comprising a combination of abdominal ultrasonography with multiple biomarkers is recommended in patients with chronic liver disease, particularly in those with cirrhosis derived from advanced fibrosis. However, recent studies have shown that 1/4 to 1/3 of patients with NASH develop HCC without cirrhosis [9, 10]. Furthermore, we previously reported that serum AFP is not significantly increased in most patients with NASH-HCC [10]. In contrast, serum IgM-free apoptosis inhibitor of macrophage (AIM) is a useful biomarker of NASH-HCC, irrespective of AFP or DCP status [11].

AIM, a secretory glycoprotein produced by macrophages, suppresses macrophage apoptosis [12]. AIM comprises three scavenger receptor cysteine-rich domains with a molecular weight of approximately 40 kDa. In blood, AIM combines with high-molecular weight IgM pentamers at a 1:1 equimolar ratio through a disulfide bond and a charge-based interaction between AIM and IgM Fc, and exists in a stabilized, inactive state [13, 14]. However, it exhibits various biological activities as IgM-free AIM, following its release from IgM [15]. For instance, animal experiments have demonstrated its lipolytic activity in adipocytes and hepatocytes, anti-liver cancer activity based on activation of complement-dependent cytotoxicity, and cell residue removal activity in renal tubules [16–18]. Thus, AIM may be considered a therapeutic target for obesity, fatty liver, liver cancer, and acute kidney injury. In the liver, AIM is taken up by cells via CD36 where it demonstrates lipolytic activity in normal cells; however, in cancer cells, cellular uptake is suppressed, and AIM accumulates on the cell surface, activating complement proteins and causing subsequent cancer cell death [17].

To use AIM as a tumor marker, it is necessary to establish a method capable of specifically measuring activated IgM-free AIM without interference from IgM-bound AIM in blood. Although an enzyme-linked immunosorbent assay (ELISA) was used for IgM-free AIM [11, 19], a higher-throughput method is required considering large-scale utilization in clinical settings. To this end, we developed an antibody specific to IgM-free AIM and established a fully automatic and high-throughput electrochemiluminescence immunoassay (ECLIA) method that can specifically measure IgM-free AIM. Using this method, we examined the utility of IgM-free AIM as a diagnostic biomarker for HCC in each group of etiologies causing HCC by analyzing serum samples collected from patients with NASH, hepatitis B virus (HBV), and hepatitis C virus (HCV). Furthermore, we established the ECLIA method capable of measuring both IgM-free AIM and IgM-bound AIM (total AIM); we also investigated the existing AIM form in blood by measuring the fractionated samples from the sera of patients with NASH and NASH-HCC using size-exclusion chromatography to investigate the clinical significance of IgM-free AIM in NASH-HCC.

## Materials and methods

### Subjects

In total, 212 patients with liver disease, 117 without malignancy, and 95 with HCC who visited Saiseikai Suita Hospital from January 2010 to July 2017 were included in the study (Table 1). The study protocol was approved by the Human Ethics Committee of Saiseikai Suita Hospital. Informed consent was obtained from all patients, and the study protocol was performed in accordance with the Declaration of Helsinki. The serum samples of cancers other than liver cancer were provided by the Tsukuba Medical Laboratory of Education and Research Center and University of Tsukuba Hospital in Japan in accordance with the “Guidelines for clinical measurement and diagnosis technology improvement project,” promoted by the same institutes. In addition to these specimens, serum samples were obtained from healthy volunteers who were employees of Sekisui Medical Co. (Tokyo, Japan). Written informed consent was obtained from all volunteers at the time of enrollment, in accordance with the code of ethics of Sekisui Medical Co.

**Table 1:** List of specimens used in this study

	Non-HCC (hepatitis and cirrhosis)	HCC tumor stage				Total
		T1	T2	T3	T4	
HBV						
Number	19	17	7	1	2	46
Sex (m/f)	12/7	22/5				
Age^a^ (years)	43.8 ± 8.9	64.5 ± 9.8				
Percentage of patients with chronic hepatitis to all non-HCC/HCC patients	94.7%	92.6%				
Percentage of patients with liver cirrhosis to all non-HCC/HCC patients	0.0%	7.4%				
Platelet^a^ (10^10^/L)	17.9 ± 4.3	15.0 ± 4.9				
Type IV collagen^a^ (µg/L)	4.8 ± 1.1^b^	6.7 ± 2.5^c^				
FIB-4 index^a^	1.92 ± 1.34	2.89 ± 1.01				
HCV						
Number	55	27	7	7	0	96
Sex (m/f)	25/30	29/12				
Age^a^ (years)	67.4 ± 12.9	72.6 ± 8.1				
Percentage of patients with chronic hepatitis to all non-HCC/HCC patients	78.2%	100.0%				
Percentage of patients with liver cirrhosis to all non-HCC/HCC patients	21.8%	0.0%				
Platelet^a^ (10^10^/L)	13.2 ± 4.5	12.8 ± 5.9				
Type IV collagen^a^ (µg/L)	6.7 ± 1.8^d^	8.0 ± 1.4^e^				
FIB-4 index^a^	4.08 ± 2.32	4.93 ± 2.92				
NASH						
Number	43	11	8	3	5	70
Sex (m/f)	15/28	14/13				
Age^a^ (years)	60.9 ± 14.0	72.2 ± 9.8				
Percentage of patients with chronic hepatitis to all non-HCC/HCC patients	72.1%	46.4%				
Percentage of patients with liver cirrhosis to all non-HCC/HCC patients	9.3%	53.6%				
Platelet^a^ (10^10^/L)	20.0 ± 7.7	13.9 ± 5.1				
Type IV collagen^a^ (µg/L)	6.2 ± 1.9^f^	8.4 ± 2.0^ g^				
FIB-4 index^a^	2.68 ± 1.66	5.04 ± 3.26				
Total						212

*HBV* hepatitis B virus, *HCC* hepatocellular carcinoma, *HCV* hepatitis C virus, *NASH* non-alcoholic steatohepatitis

^a^Age, Platelet, Type IV collagen, and FIB-4 index are shown as mean ± standard deviation

^b^Value for 18 patients (data of 1 patient that is missing was not considered)

^c^Value for 25 patients (data of 2 patients that are missing were not considered)

^d^Value for 54 patients (data of 1 patient that is missing was not considered)

^e^Value for 36 patients (data of 5 patients that are missing were not considered)

^f^Value for 42 patients (data of 1 patient is missing was not considered)

^g^Value for 24 patients (data of 3 patients that are missing were not considered)

### Clinical and laboratory assessment

Blood samples were obtained from the subjects in the morning after an overnight fast within 2 weeks prior to the liver biopsy and before each blood test. Clinical laboratory tests were conducted at the Department of Clinical Laboratory at Saiseikai Suita Hospital.

### Histopathological examination

Liver biopsies were performed with a 16-gauge aspiration needle (Hakko Co., Ltd., Nagano, Japan), yielding specimens of at least 2.0 cm in length. The specimens were fixed in formalin, embedded in paraffin, and subjected to hematoxylin–eosin, Masson’s trichrome, and Perl’s iron staining. Histological assessment was performed by an expert hepatologist. Patients with NAFLD were classified into four types according to Matteoni’s classification [5]: type 1, simple steatosis; type 2, steatosis with lobular inflammation; type 3, type 2 plus ballooned hepatocytes; and type 4, presence of either Mallory–Denk bodies or fibrosis. Types 1 and 2 were classified as NAFL, and types 3 and 4 were classified as NASH. Furthermore, patients with NAFLD and fibrosis but without ballooning hepatocytes were classified as type 4. HCV infection was diagnosed based on the detection of HCV RNA using the Cobas 6800/8800 systems (Roche Molecular Systems Inc., CA, USA). HBV infection was diagnosed using an automated immunoassay analyzer LUMIPULSE (Fujirebio, Inc., Tokyo, Japan). HCC was diagnosed by histological examination or findings from ultrasound sonography, computed tomography (CT), magnetic resonance imaging (MRI), or hepatic angiography. Vascular invasion was assessed using dynamic CT, MRI, or angiography. DCP and AFP levels were measured in all patients. We used the optimal cut-off points of DCP and AFP in the analysis of HCC according to the manufacturer’s instructions.

### Size-exclusion chromatography

Size-exclusion chromatography of serum samples was conducted using a Prominence high-performance liquid chromatograph (HPLC) (Shimadzu Co., Ltd., Tokyo, Japan) with a G3000SW_XL_ column (Tosoh Co., Tokyo, Japan) at a flow rate of 1.0 mL/min with 50 mM phosphate-buffered saline (pH 7.4). The fractionated samples were collected in 500 µL aliquots.

### Preparation of anti-AIM monoclonal antibody

To obtain anti-AIM monoclonal antibodies, BALB/c mice were immunized three times using full-length recombinant AIM with an adjuvant. Then, mouse spleen cells and myeloma cells were fused as previously described [20]. Antibody-producing clones that reacted with recombinant AIM and IgM-free AIM in the serum samples from patients with NASH-HCC were screened using ELISA and western blotting, followed by cloning using the limiting dilution method. The established clonal cells were injected into the abdominal cavity of BALB/cAJcL-nu/nu nude mice, and the IgG fraction was purified from the ascites fluid. One clonal cell line (No. 12) that produced antibodies specific to IgM-free AIM and two clonal cell lines (No. 8 and 11) that reacted with both IgM-free and IgM-bound AIM were selected with the ECLIA method described below.

### Preparation of ECLIA reagents

Anti-AIM antibody No. 12 or 11 was mixed with magnetic beads with epoxy groups and incubated at 25 °C for 24–72 h to coat the beads with antibodies. The beads were then washed and blocked with Tris buffer (pH 7.8) containing 0.1% BSA at 25 °C for 24 h. Finally, the beads were washed with blocking buffer and stored at 4 °C. Ruthenium (Ru)-labeled anti-AIM antibody No. 8 or 11 was prepared as follows. First, Ru with N-hydroxysuccinimide residue (Origin Tag-NHS ESTER, IGEN, USA) was added to the anti-AIM antibody No. 8 or 11 solution and incubated at 25 °C for 30 min. Then, 2 M glycine solution was added and incubated for 20 min to stop the reaction. Ru-labeled anti-AIM antibody No. 8 or 11 was subjected to gel filtration column chromatography (Sephadex G-25, GE Healthcare, Inc., USA) to remove unlabeled Ru.

### Measurement of IgM-free AIM, IgM-bound AIM, and IgM using ECLIA

IgM-free AIM, IgM-bound AIM, and IgM were measured using the ECLIA on a Picolumi III automatic analyzer (Sekisui Medical Co., Japan). Briefly, for measuring IgM-free AIM, 5 µL serum sample was diluted with 100 µL reaction buffer containing mouse IgG (200 µg/mL); the diluted sample was then incubated with 25 µL magnetic beads coated with anti-AIM antibody No. 12 at 30 °C for 8 min (first reaction). When the HPLC fractionated sample was measured, 100 µL undiluted fraction was used. After the first reaction, the beads were washed, and 100 µL Ru-labeled anti-AIM antibody No. 11 was added and the mixture was incubated at 30 °C for 8 min (second reaction).

The beads were then washed to remove the unreacted Ru-labeled antibody to form an electrode along with the electrolyte solution; the photons emitted from ruthenium by the electrochemical reaction were measured using a photomultiplier. The concentration of IgM-free AIM (µg/mL) was calculated based on the calibration curve from the photon count of the AIM standard concentrations (0.1, 1, 4, and 8 µg/mL). The AIM standard was obtained by affinity purification from the supernatant of HEK293 cells expressing full-length recombinant AIM, using a Sepharose column immobilized with the anti-AIM antibody No. 12. The purified recombinant AIM was freeze-dried and weighed, and the concentration of each standard was determined based on the weight of the freeze-dried sample. When measuring both IgM-free AIM and IgM-bound AIM, magnetic beads coated with both anti-AIM antibody No. 11 and Ru-labeled anti-AIM antibody No. 8 were used for ECLIA as described above. To measure IgM, magnetic beads coated with commercially available anti-IgM monoclonal antibody (ADTEC Co., Japan) and the same antibody labeled with Ru were used for ECLIA.

### Receiver operating characteristic (ROC) curves for assessing the diagnostic accuracy of AIM

ROC curves were obtained by calculating the sensitivity and specificity of the assay at every possible cut-off point and plotting sensitivity against [1-specificity] in SPSS for Windows Version 24 (SPSS Japan, Tokyo, Japan). The area under the ROC curve (AUROC) was calculated to determine the diagnostic accuracy of the assay. Appropriate cut-off points were examined for balancing the sensitivity and specificity of the ROC curve, and the optimal cut-off point was identified as that yielding the minimal value for [(1 − sensitivity)^2^ + (1 − specificity)^2^] or the maximal value for [sensitivity + specificity − 1] [21].

### Statistical analyses

Statistical analysis was performed using IBM SPSS Statistics Version 24 for Windows. All values are expressed as the mean ± standard deviation. Differences in mean values between groups were assessed using the Mann–Whitney U-test, whereas correlations between parameters were evaluated using Pearson’s correlation coefficient. For all analyses, statistical significance was set at *p* < 0.05.

## Results

### Analytical performance of the newly established ECLIA specific for IgM-free AIM

#### Evaluation of specificity for IgM-free AIM

**Fig. 1:**
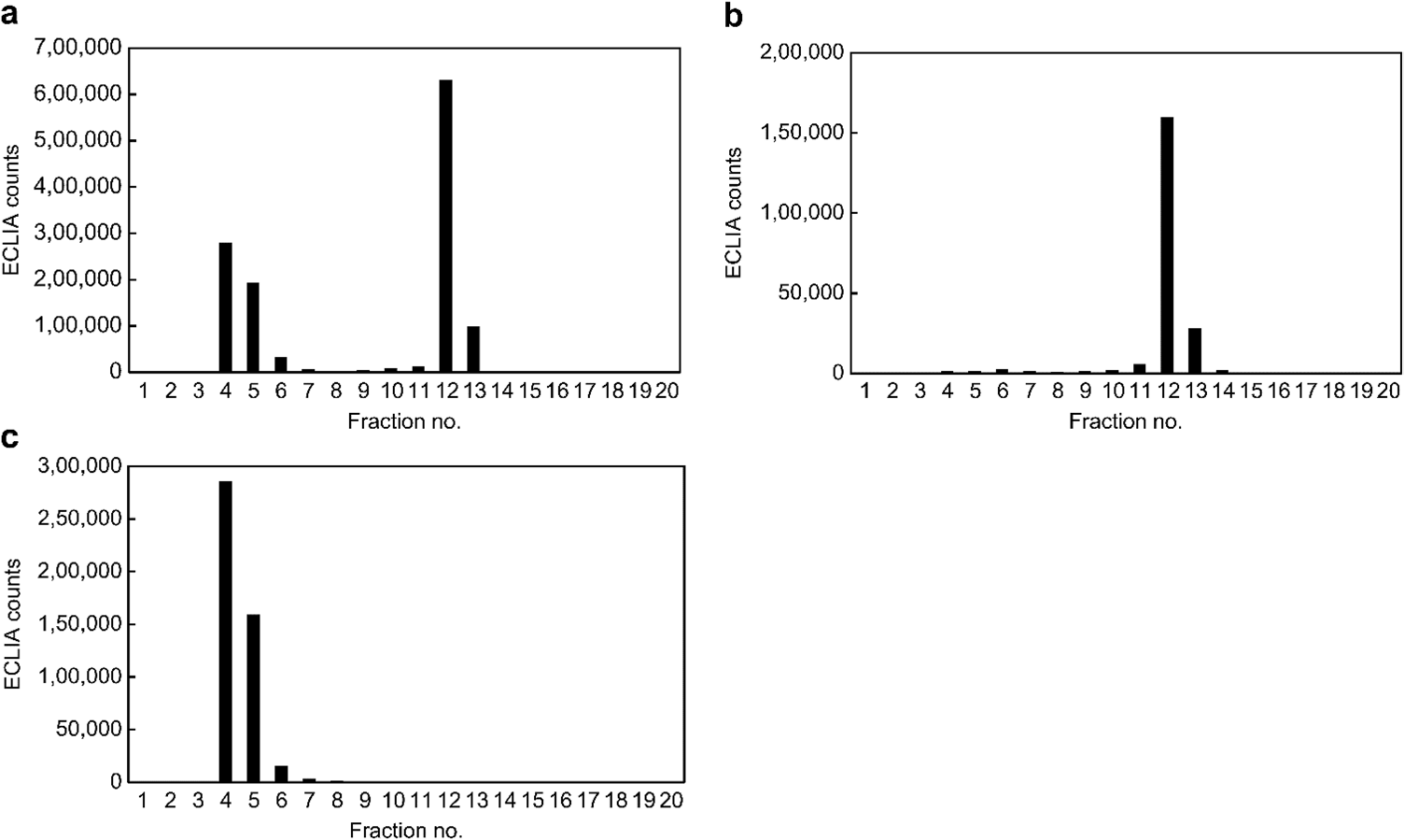
ECLIA counts of size-exclusion chromatography fractionated samples from the serum of one patient with NASH-HCC. The results of ECLIA for **a** IgM-free AIM and IgM-bound AIM, **b** IgM-free AIM, and **c** IgM alone. **a** Bimodal peaks of ECLIA counts observed in fractions No. 4–6 and 12–13. **b** One peak of ECLIA counts observed in fractions No. 12–13. **c** One peak of ECLIA counts observed in fractions No. 4–6. *AIM* apoptosis inhibitor of macrophage, *ECLIA* electrochemiluminescence immunoassay, *HCC* hepatocellular carcinoma, *NASH* non-alcoholic steatohepatitis

To evaluate the analytical performance of ECLIA, size-exclusion chromatography fractionated samples from the serum of one patient with NASH-HCC were assayed by the ECLIA using magnetic beads coated with anti-AIM antibody No. 11 and Ru-labeled anti-AIM antibody No. 8 to measure IgM-free AIM and IgM-bound AIM. Bimodal peaks of ECLIA counts were observed in fractions No. 4–6 and No. 12–13 (Fig. 1a). In contrast, for IgM-free AIM-specific ECLIA using beads coated with anti-AIM antibody No. 12 and Ru-labeled anti-AIM antibody No. 11, only one peak of ECLIA counts was observed in fractions No. 12–13 (Fig. 1b). Furthermore, using ECLIA to measure IgM, only one peak of ECLIA counts was observed in fractions No. 4–6 (Fig. 1c). These data indicate that IgM-bound AIM and IgM-free AIM were fractionated in fractions No. 4–6 and No. 12–13, respectively, and that ECLIA using beads coated with anti-AIM antibody No. 12 and Ru-labeled anti-AIM antibody No. 11 specifically measures IgM-free AIM, but not IgM-bound AIM.

Next, 25 serum samples from healthy individuals (*n* = 5) and patients with NAFL (*n* = 5), NASH (*n* = 5), and NASH-HCC (*n* = 10) were fractionated by size-exclusion chromatography, and both IgM-bound AIM and IgM-free AIM were measured by ECLIA using beads coated with anti-AIM antibody No. 11 and Ru-labeled anti-AIM antibody No. 8. Furthermore, the same serum samples were directly measured for IgM-free AIM concentration by ECLIA using anti-AIM antibody No. 12 and Ru-labeled anti-AIM antibody No. 11 to investigate its correlation with the fractionated IgM-free AIM samples by size-exclusion chromatography. The sum of ECLIA counts from fraction No. 12–13 by size-exclusion chromatography of the 25 serum samples and directly measured IgM-free AIM concentration were well-correlated (*r* = 0.98, *p* < 0.0001; Online Resource 1), indicating that ECLIA using anti-AIM antibody No. 12 and Ru-labeled anti-AIM antibody No. 11 specifically and accurately measures the IgM-free AIM concentration in serum samples.

#### Evaluation of accuracy for IgM-free AIM measurement

To evaluate the sample dilution linearity, two serum samples from patients with NASH-HCC (No. 1 and 2) were serially diluted from 1:1 to 1:64 and IgM-free AIM was measured by ECLIA using beads coated with anti-AIM antibody No. 12 and Ru-labeled anti-AIM antibody No. 11. Good linearity was observed for each serum sample (Online Resource 2a and b).

Next, assay repeatability was evaluated by five consecutive assays of IgM-free AIM in three serum samples, and the intra-assay coefficients of variation were 0.5–4.0%. Furthermore, assay reproducibility was evaluated by assaying three serum samples on four different days, and the inter-day assay coefficients of variation were 1.8–3.2%. Both the intra-assay and the inter-day assay coefficients of variation obtained indicated good precision.

Finally, the effect of interfering substances was examined by spiking the serum samples with Interference Check A Plus and RF Plus (Sysmex, Japan) and measuring the IgM-free AIM using ECLIA. No change in the IgM-free AIM level was observed in serum samples spiked with bilirubin F and C up to 25 mg/dL, hemoglobin up to 500 mg/dL, chyle up to 3,000 FTU, and rheumatoid factor (RF) up to 500 IU/mL (Online Resource 3), indicating that the assay was not affected by interfering substances.

### IgM-free AIM concentration in sera from patients with liver disease

**Fig. 2:**
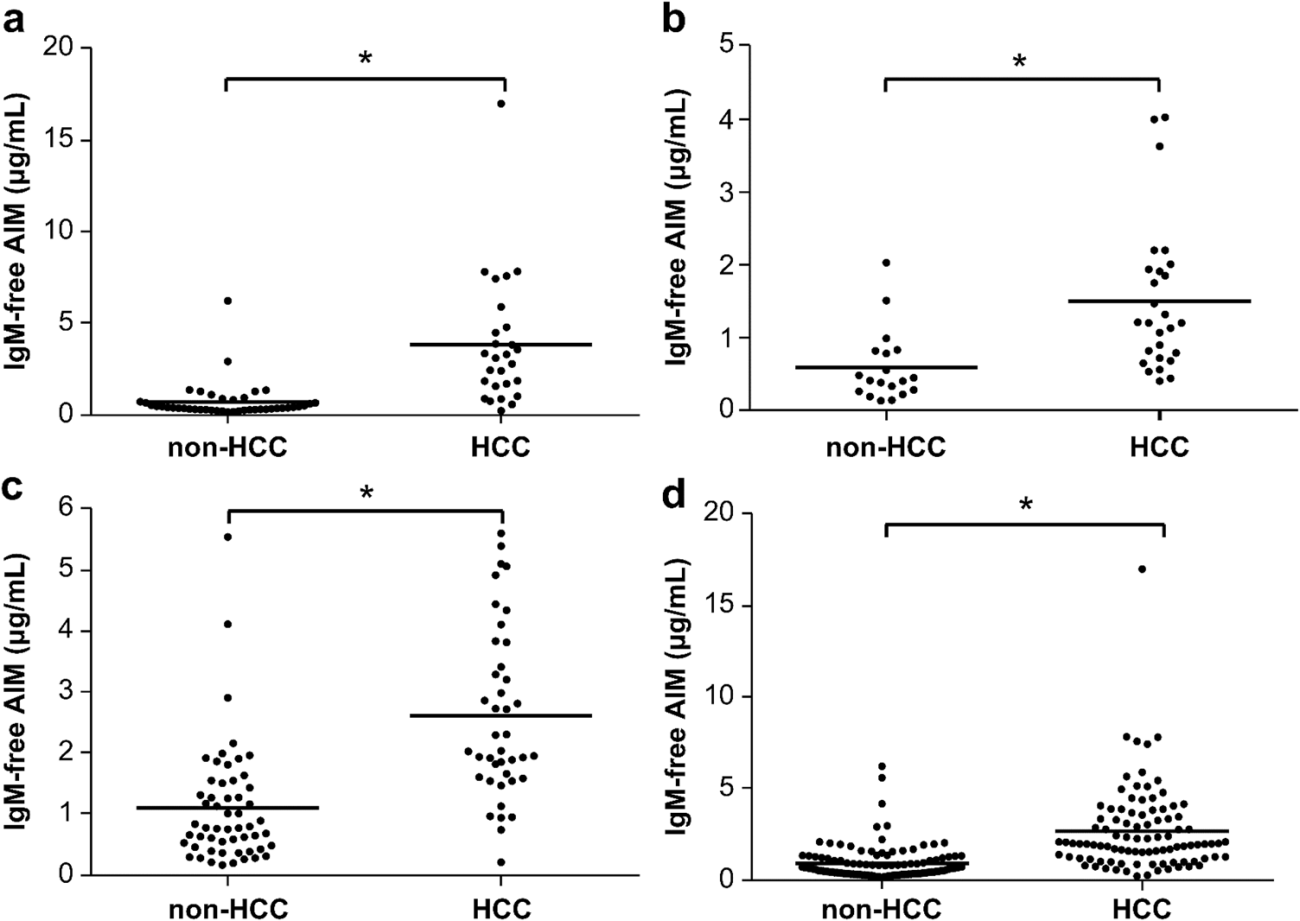
Distribution mapping of serum IgM-free AIM level in non-HCC (hepatitis and cirrhosis) and HCC patient groups for all cancer stages. The results are shown in **a**–**d** are for all cancer stages of NASH-HCC, HBV-HCC, HCV-HCC, and all the patients, respectively. The serum IgM-free AIM level was significantly higher in the HCC patient group than that in the non-HCC patient group (*p* < 0.001) regardless of the pathogenesis. *p < 0.001 (Mann–Whitney U-test). *AIM* apoptosis inhibitor of macrophage, *HBV* hepatitis B virus, *HCC* hepatocellular carcinoma, *HCV* hepatitis C virus, *NASH* non-alcoholic steatohepatitis

**Fig. 3:**
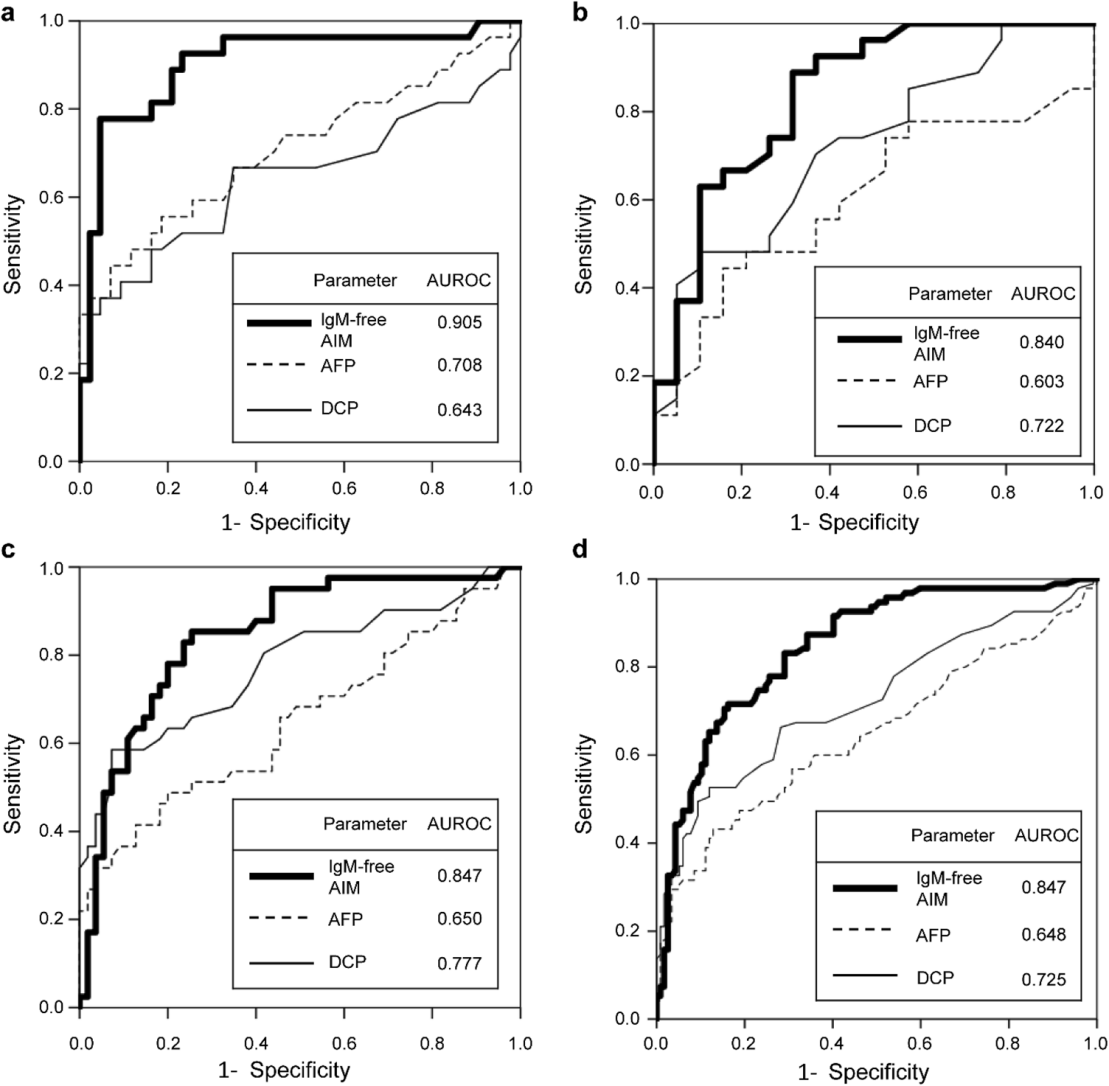
ROC analyses of IgM-free AIM, AFP, and DCP for all cancer stages. The sensitivity and specificity of the markers were determined to discriminate the patients with HCC from those without HCC. **a–d** Include all cancer stages of NASH-HCC, HBV-HCC, HCV-HCC, and all the patients, respectively. IgM-free AIM showed the highest AUROC for each disease group and all the patients. *AFP* alpha-fetoprotein, *AIM* apoptosis inhibitor of macrophage, *DCP* des-γ-carboxy prothrombin, *HBV* hepatitis B virus, *HCC* hepatocellular carcinoma, *HCV* hepatitis C virus, *NASH* non-alcoholic steatohepatitis, *ROC* receiver operating characteristic, *AUROC* area under the receiver operating characteristic

To evaluate the diagnostic value of IgM-free AIM, sera from the non-HCC (chronic hepatitis and cirrhosis) patient group and the HCC patient group including all cancer stages related to NASH, HCV, and HBV were measured with ECLIA using the anti-AIM antibody No. 12 and No. 11. The IgM-free AIM value was significantly higher in the HCC patient group than in the non-HCC patient group (*p* < 0.001; Fig. 2a–d). The same result was obtained when patients with HCC were limited to stages 1–2 (Online Resource 4). Furthermore, we compared the ROC curves of IgM-free AIM calculated from specificity and sensitivity with those of the conventional HCC biomarkers, AFP and DCP, for each liver disease related to NASH, HCV, HBV, and for all the patients. IgM-free AIM showed the highest AUROC in each disease group including all cancer stages of HCC and in all the patients together (Fig. 3a–d); the same result was obtained when HCC was limited to stages 1–2 (Online Resource 5). After balancing the sensitivity and specificity of the ROC curve calculated from all non-HCC groups (*n* = 117) and all HCC groups (*n* = 95), the optimal cut-off of serum IgM-free AIM for predicting HCC was estimated to be 1.6 µg/mL as it showed the maximal diagnostic accuracy. Using this cut-off point, the sensitivity, specificity, and accuracy of IgM-free AIM were determined and compared with those of AFP and DCP, which were set to 20 ng/mL and 40 mAU/mL, respectively. The accuracy of IgM-free AIM for HCC detection related to NASH, HBV, and HCV was 87.1%, 60.9%, and 83.3%, respectively, and that in all the patients was 79.7%, indicating that IgM-free AIM has a higher clinical accuracy than AFP or DCP (Table 2). The same result was obtained when HCC was limited to stages 1–2 (Online Resource 6). IgM-free AIM is produced by macrophages, whereas DCP and AFP are produced by liver cancer cells [22, 23]; therefore, a combination assay of IgM-free AIM with AFP or DCP may be useful for HCC diagnosis. We then investigated the correlation between IgM-free AIM and AFP or DCP. IgM-free AIM did not show a definite correlation with AFP (*r* = 0.224, *p* = 0.001; Online Resource 7a) or DCP (*r* = 0.139, *p* = 0.043; Online Resource 7b). Furthermore, due to its higher positivity rate, IgM-free AIM alone can be a more sensitive marker for HCC than AFP or DCP alone, respectively (63.2% vs 33.7–38.9%; Online Resource 8a–c). In addition, we compared the positivity rate in the combination of IgM-free AIM and AFP or DCP with that in the combination of AFP and DCP. The positivity rates in the combination of IgM-free AIM and AFP, and that of IgM-free AIM and DCP were 74.7% and 77.9%, respectively, indicating that the positivity rates of IgM-free AIM in combination with AFP or DCP are higher than those in the combination of AFP and DCP (49.5%; Online Resource 8d–f). It is notable that IgM-free AIM alone showed a higher sensitivity than the combination of AFP and DCP.

**Table 2:** Comparison of the sensitivity, specificity, and accuracy of IgM-free AIM, AFP, and DCP for discriminating HCC of all cancer stages

	Marker	Sensitivity (%)	Specificity (%)	Accuracy (%)
Comparison of diagnostic performance for NASH-HCC				
All cancer stages	IgM-free AIM	74.1	95.3	87.1
	AFP	18.5	100	68.6
	DCP	40.7	90.7	71.4
Comparison of diagnostic performance for HBV-HCC				
All cancer stages	IgM-free AIM	37.0	94.7	60.9
	AFP	33.3	84.2	54.3
	DCP	33.3	94.7	58.7
Comparison of diagnostic performance for HCV-HCC				
All cancer stages	IgM-free AIM	87.8	80.0	83.3
	AFP	41.5	87.3	67.7
	DCP	41.5	96.4	72.9
Comparison of diagnostic performance for all HCC patients				
All cancer stages	IgM-free AIM	69.5	88.0	79.7
	AFP	32.6	91.5	65.1
	DCP	38.9	94.0	69.3

The cut-off values of IgM-free AIM, AFP, and DCP were set at 1.6 µg/mL, 20 ng/mL, and 40 mAU/mL, respectively

*AFP* alpha-fetoprotein, *AIM* apoptosis inhibitor of macrophage, *DCP* des-γ-carboxy prothrombin, *HBV* hepatitis B virus, *HCC* hepatocellular carcinoma, *HCV* hepatitis C virus, *NASH* non-alcoholic steatohepatitis

### IgM-free AIM levels in cancers other than liver cancer, and the relationship of IgM-free AIM between cancer stage and age

To explore whether IgM-free AIM is elevated specifically in patients with liver cancer, we also measured IgM-free AIM levels in patients with different types of cancer. IgM-free AIM was significantly high only in patients with liver cancer and was not elevated in patients with other types of cancer compared to normal volunteers (Online Resource 9). On the other hand, there was no difference in IgM-free AIM levels between patients with different cancer stages (Online Resource 10), and no correlation was seen between IgM-free AIM and age regardless of the presence of HCC and non-HCC (Online Resource 11), indicating that the stage of cancer and the age are independent of IgM-free AIM level.

### Measurement of IgM-free and IgM-bound AIM in fractionated serum samples by size-exclusion chromatography

**Fig. 4:**
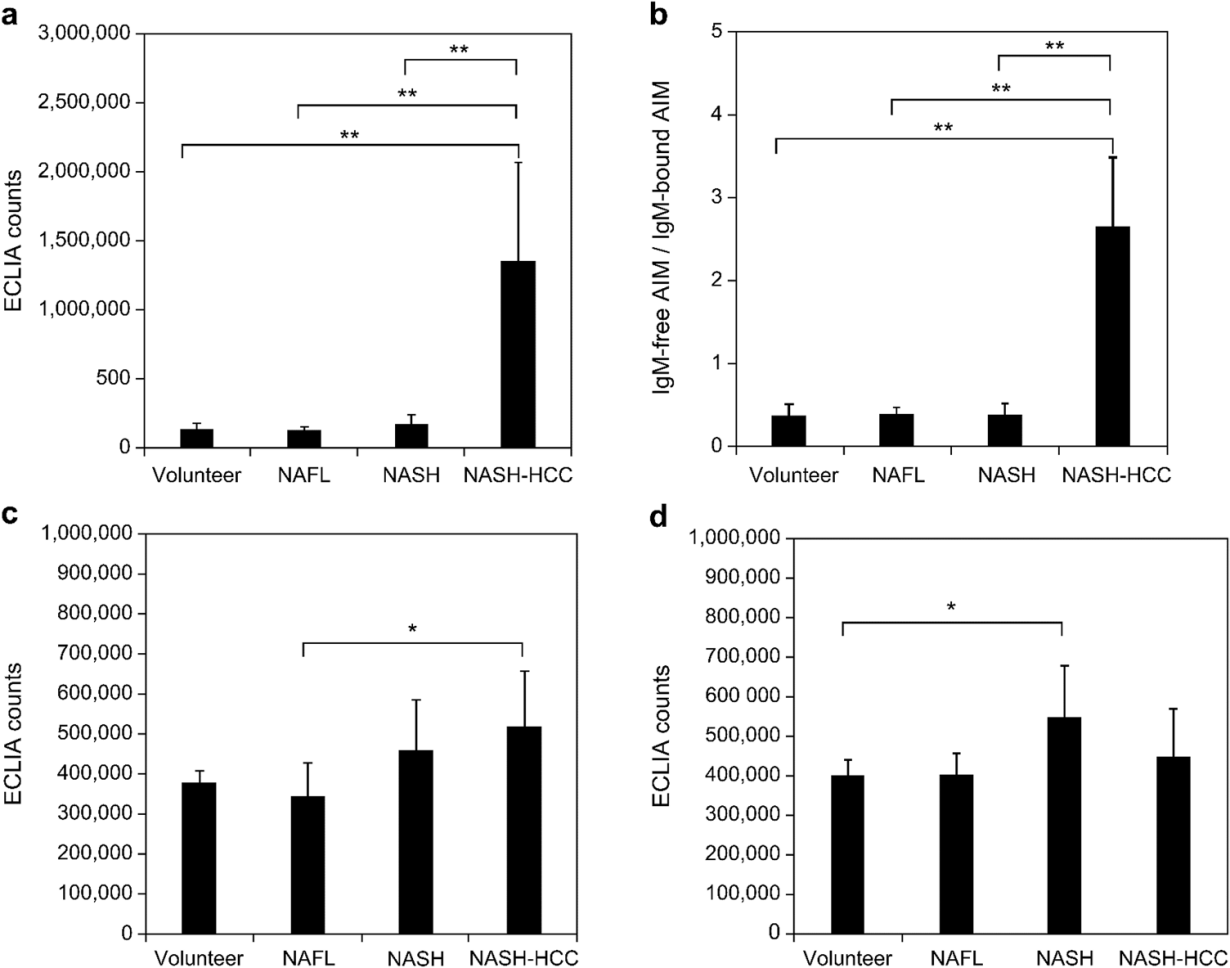
ECLIA counts of size-exclusion chromatography fractionated serum samples from healthy volunteers and from patients with NAFL, NASH, and NASH-HCC. The mean values of ECLIA counts for **a** IgM-free AIM, **b** IgM-free AIM/IgM-bound AIM ratio, **c** IgM-bound AIM, and **d** IgM determined from the fractionated samples in each disease (25 serum samples from healthy volunteers (*n* = 5), and from patients with NAFL (*n* = 5), NASH (*n* = 5), and NASH-HCC (*n* = 10)). **a** IgM-free and **b** IgM-free AIM/IgM-bound AIM ratio were significantly higher in patients with NASH-HCC. **c** IgM-bound AIM and **d** IgM were relatively higher in patients with NASH and NASH-HCC. **p < 0.001, *p < 0.05 (Mann–Whitney U-test). *AIM* apoptosis inhibitor of macrophage, *ECLIA* electrochemiluminescence immunoassay, *HCC* hepatocellular carcinoma, *NAFL* non-alcoholic fatty liver, *NASH* non-alcoholic steatohepatitis

**Fig. 5:**
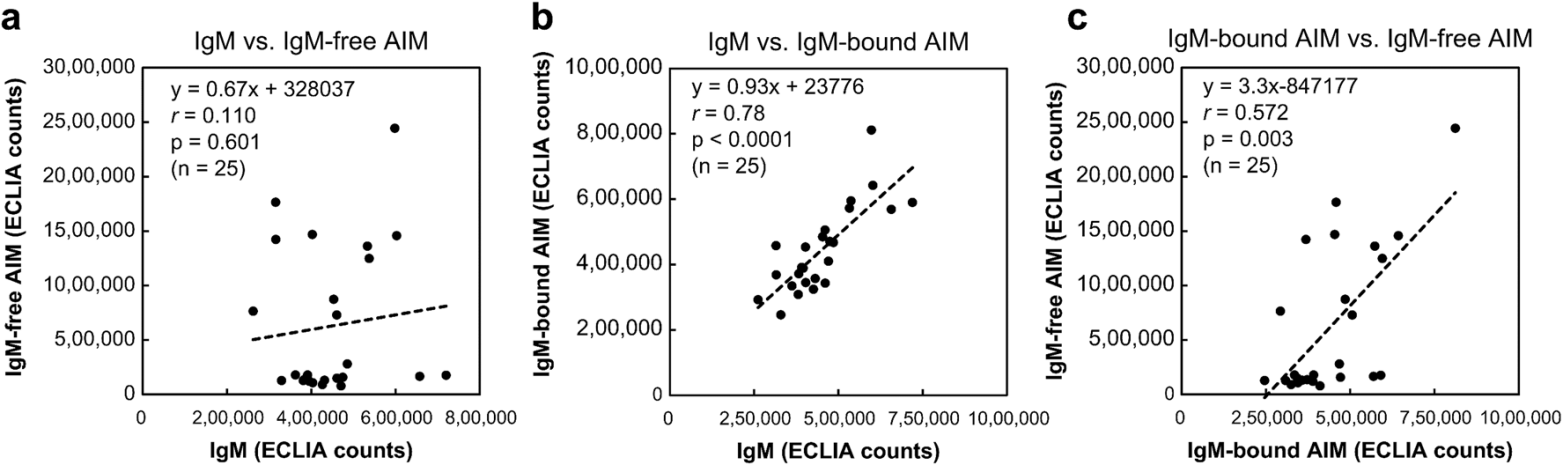
Correlations of ECLIA counts among IgM, IgM-bound AIM, and IgM-free AIM fractions. **a** No significant correlation was observed between the ECLIA counts of IgM and IgM-free AIM. **b** ECLIA counts of IgM and IgM-bound AIM showed a significant positive correlation. **c** ECLIA counts of IgM-bound and IgM-free AIM showed a weak positive correlation. *AIM* apoptosis inhibitor of macrophage, *ECLIA* electrochemiluminescence immunoassay

Considering that patients with HCC showed higher positivity for IgM-free AIM in their sera, we investigated the existing form of AIM in blood. Therefore, the sera from healthy volunteers and patients with NAFL, NASH, and NASH-HCC were fractionated by size-exclusion chromatography, and the fractionated samples were assayed for IgM-bound AIM and IgM-free AIM by ECLIA using beads coated with anti-AIM antibody No. 11 and Ru-labeled anti-AIM antibody No. 8. AIM antigen activity was observed in fractions No. 4–6 (IgM-bound AIM) and fractions No. 12–13 (IgM-free AIM) from all patients (Online Resource 12). Interestingly, the peaks of fractions No. 4–6 (IgM-bound AIM) from healthy volunteers and patients with NAFL and NASH were higher than the peaks of fractions No. 12–13 (IgM-free AIM), whereas the peaks of fractions No. 12–13 (IgM-free AIM) from patients with NASH-HCC were higher than the peaks of fractions No. 4–6 (IgM-bound AIM). Subsequently, 25 serum samples from healthy volunteers (*n* = 5) and patients with NAFL (*n* = 5), NASH (*n* = 5), and NASH-HCC (*n* = 10) were fractionated in the same manner and assayed for IgM-bound AIM, IgM-free AIM, and IgM by ECLIA. The IgM-free AIM counts and IgM-free/IgM-bound AIM count ratios were significantly higher in the NASH-HCC group than in the other groups (*p* < 0.001; Fig. 4a, b). The IgM-bound AIM counts of patients with NASH-HCC did not significantly increase compared with those of healthy volunteers and patients with NASH; however, a weak, yet statistically significant, increase was observed compared with the NAFL group (*p* = 0.028; Fig. 4c). The IgM counts of patients with NASH-HCC were not significantly higher than those of patients in the other groups (*p* = 0.032; Fig. 4d). Furthermore, we evaluated the correlations of ECLIA counts among IgM-free AIM, IgM-bound AIM, and IgM in all serum samples. IgM-bound AIM correlated well with IgM (*r* = 0.78, *p* < 0.0001), whereas IgM-free-AIM did not (*r* = 0.110, *p* = 0.601; Fig. 5a, b). There was a weak positive correlation between IgM-bound AIM and IgM-free-AIM (*r* = 0.572, *p* = 0.003; Fig. 5c). These data indicate that IgM-free AIM counts are specifically increased in the blood of patients with NASH-HCC because of dissociation from IgM.

## Discussion

AIM plays a key role in protecting liver cells from lipid accumulation and carcinogenesis, and the release of free AIM from IgM is essential for exerting its biological function [11, 17, 24]. Therefore, IgM-free AIM is considered a candidate diagnostic marker for liver diseases. Moreover, serum IgM-free AIM levels are a sensitive diagnostic marker for NASH-HCC [11].

In this study, we established a novel high-throughput method, ECLIA, that enables us to automatically measure the IgM-free AIM levels, and verified its specificity for IgM-free AIM. It specifically and precisely measured IgM-free AIM without reacting with IgM-bound AIM in serum samples.

Serum IgM-free AIM levels were significantly higher in both the NASH-HCC patient group and the viral HCC patient group, and both ROC analysis and the diagnostic accuracy of IgM-free AIM demonstrate that serum IgM-free AIM may be a potential universal HCC diagnostic marker that could be superior to AFP or DCP, even in early cancer stages. In fact, a mouse carcinogenicity study indicated that AIM begins accumulating on the hepatic cell surface at the preneoplastic stage to exert an anti-HCC effect through complement activation [17]. Thus, serum IgM-free AIM levels may be elevated in the early stage of HCC, even in humans. This report may also explain why some non-HCC patients showed high IgM-free AIM levels in this study, i.e., AIM released from IgM may start to eliminate cancer cells in the very early stages of HCC which cannot be diagnosed by conventional tools. This result is consistent with the previous report [11]. In fact, considering the growth rate of HCC tumors reported in a previous study [25], it may take several years until HCC lesions with an approximate diameter of 1 cm can be detected using ultrasound or computed tomography; therefore, further follow-up studies are needed to demonstrate whether non-HCC patients showing high IgM-free AIM levels already have microcarcinoma. On the other hand, there was no difference in IgM-free AIM levels between patients with different cancer stages, which may also be related to the hypothesis that AIM is released from IgM in the early stages of HCC. Furthermore, according to this study, the combination of IgM-free AIM with AFP or DCP can be more useful for the diagnosis of HCC than that of AFP and DCP. Thought to be one of the immunological parameters produced by macrophages, AIM—which is different from AFP or DCP produced by cancer tissue—may be a unique biomarker for HCC. AIM is reported to be produced mainly by liver Kupffer cells [26], and this may be the reason why IgM-free AIM increases less in other cancer types than in liver cancer. However, further research is needed to elucidate why a higher IgM-free AIM level was observed in NASH-HCC than in HBV-HCC and HCV-HCC.

As patients with HCC showed higher IgM-free AIM positivity, we sought to investigate the existing form of AIM in the blood by size-exclusion chromatography, since AIM has been shown to be associated with the IgM pentamers in blood and this association protects AIM from renal excretion [27]. Consequently, the specific increase in IgM-free AIM levels in the serum of patients with NASH-HCC was verified. Interestingly, IgM levels in these patients were significantly correlated with IgM-bound AIM levels; however, they did not correlate with IgM-free AIM levels, indicating that IgM-free AIM is a superior marker to IgM-bound AIM, as it is not influenced by IgM concentration, which can cause sex-based differences and might be influenced by liver function [27]. Although the increase in serum IgM-free AIM levels represents AIM dissociation in response to HCC occurrence, the precise mechanism underlying disease-specific dissociation remains unclear. Based on our structural analysis of the binding mode of AIM with IgM pentamer using an electron microscope [14], certain reactions that are induced upon HCC development might target the disulfide bond or the charge-based interaction between AIM and IgM Fc. However, further studies are required to elucidate the underlying mechanism.

In conclusion, we developed a clinically feasible ECLIA method for measuring IgM-free AIM in serum and demonstrated that IgM-free AIM is a more precise biomarker for HCC than the conventional biomarkers, AFP or DCP, for NASH-HCC, as well as for HBV- or HCV-related HCC, even in early cancer stages. The ECLIA method is expected to contribute to further clinical studies on AIM, which has the potential to serve as a universal biomarker for in vitro HCC diagnosis. However, due to the limited number of samples from patients with HCC available for this study, particularly those from patients in an early cancer stage, further investigation is warranted to confirm the clinical significance of IgM-free AIM as a diagnostic marker for early HCC. In addition, further study is needed to examine whether IgM-free AIM also increases in the liver malignancies that were not evaluated in this study, such as cholangiocellular carcinoma, metastatic liver cancer, and so on.

## Supplementary Information

Below is the link to the electronic supplementary material.

Supplementary file1 (PPTX 1979 kb)

Supplementary file2 (DOCX 33 kb)
