# Correction to: Hepatocellular carcinoma diagnosis using a novel electrochemiluminescence immunoassay targeting serum IgM‑free AIM

**DOI:** 10.1007/s12328-022-01601-z

**Published:** 2022-02-04

**Authors:** Tomo Shimizu, Takashi Sawada, Tomohide Asai, Yuka Kanetsuki, Jiro Hirota, Michihisa Moriguchi, Tomoaki Nakajima, Toru Miyazaki, Takeshi Okanoue

**Affiliations:** 1 Tsukuba Research Institute Research and Development Division, Sekisui Medical Co., Ltd., 3-3-1, Koyodai, Ryugasaki, Ibaraki 301-0852 Japan; 2 Department of Molecular Gastroenterology and Hepatology, Graduate School of Medical Science, Kyoto Prefectural University of Medicine, Kyoto, 602-8566 Japan; 3 Department of Hepatology, Sapporo Kosei General Hospital, Hokkaido, 060-0033 Japan; 4 Laboratory of Molecular Biomedicine for Pathogenesis, Center for Disease Biology and Integrative Medicine, Faculty of Medicine, The University of Tokyo, Tokyo, 113-0033 Japan; 5 LEAP, Japan Agency for Medical Research and Development, Tokyo, 113-0033 Japan; 6 Laboratoire d’ImmunoRhumatologie Moléculaire, Plateforme GENOMAX, Institut National de la Santé et de la Recherche Médicale UMR_S 1109, Faculté de Médecine, Fédération Hospitalo-Universitaire OMICARE, Fédération de Médecine Translationnelle de Strasbourg, Laboratory of Excellence TRANSPLANTEX, Université de Strasbourg, Strasbourg, France; 7 The Institute for AIM Medicine, Tokyo, 101-0047 Japan; 8 Department of Gastroenterology and Hepatology, Saiseikai Suita Hospital, Osaka, 564-0013 Japan

## Correction to: Clinical Journal of Gastroenterology 10.1007/s12328-021-01567-4

In the original publication of the article, the Supplementary File 2 was published with red fonts. The correct Supplementary File 2 is given in this correction.

The original article has been corrected.

## Supplementary Information

Below is the link to the electronic supplementary material.

Supplementary file1 (DOCX 29 kb)

