# Supplementary material for: Hepatocellular carcinoma diagnosis using a novel electrochemiluminescence immunoassay targeting serum IgM-free AIM
Source: Clin J Gastroenterol. 2022 Jan 4;15(1):41–51. doi: 10.1007/s12328-021-01567-4 (PMC8858287; doi:10.1007/s12328-021-01567-4)
Supplement: Supplementary file 1 — Supplementary file1 (PPTX 1979 kb) [file 12328_2021_1567_MOESM1_ESM.pptx]

## Slide 1
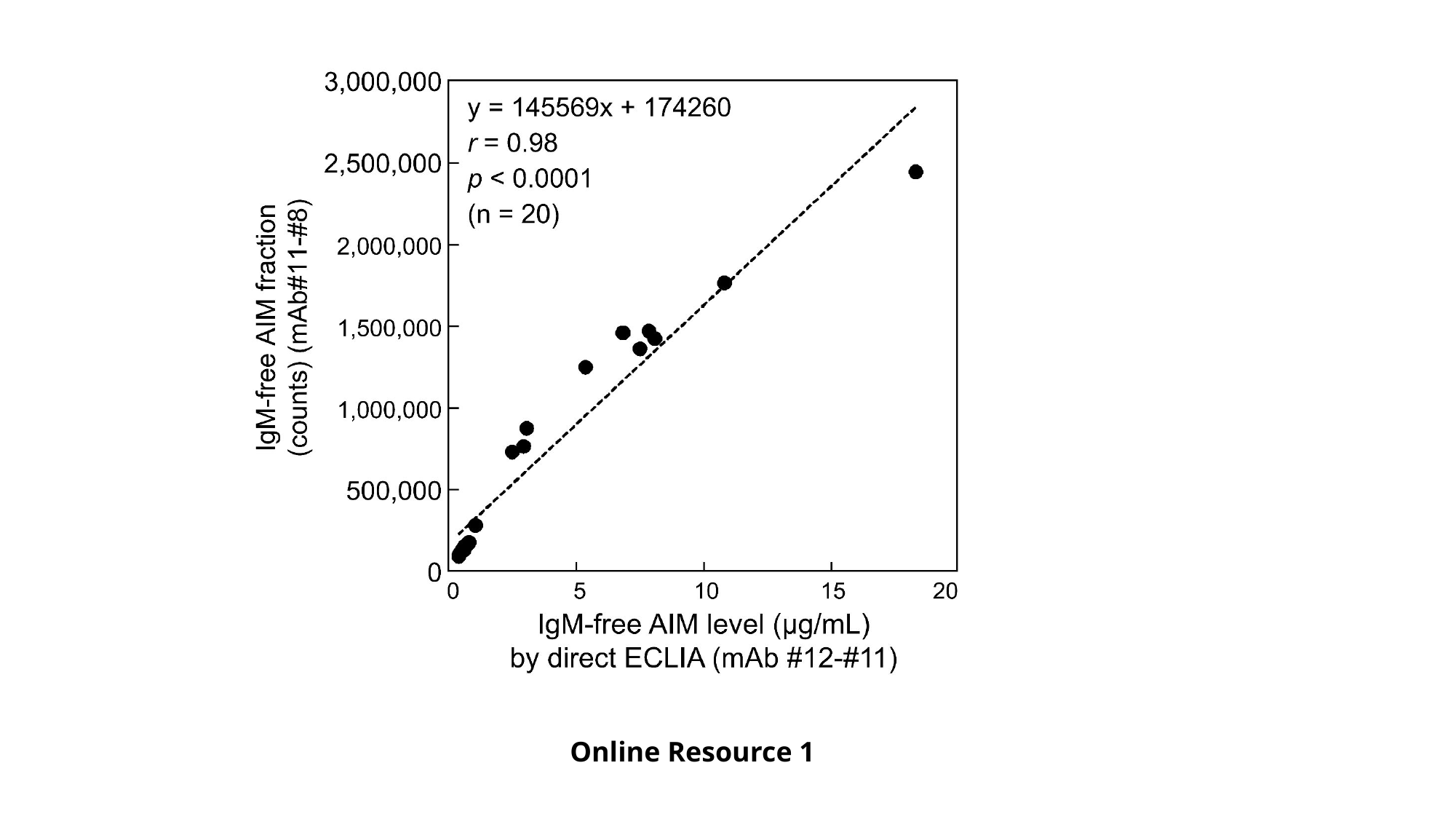

Online Resource 1

## Slide 2
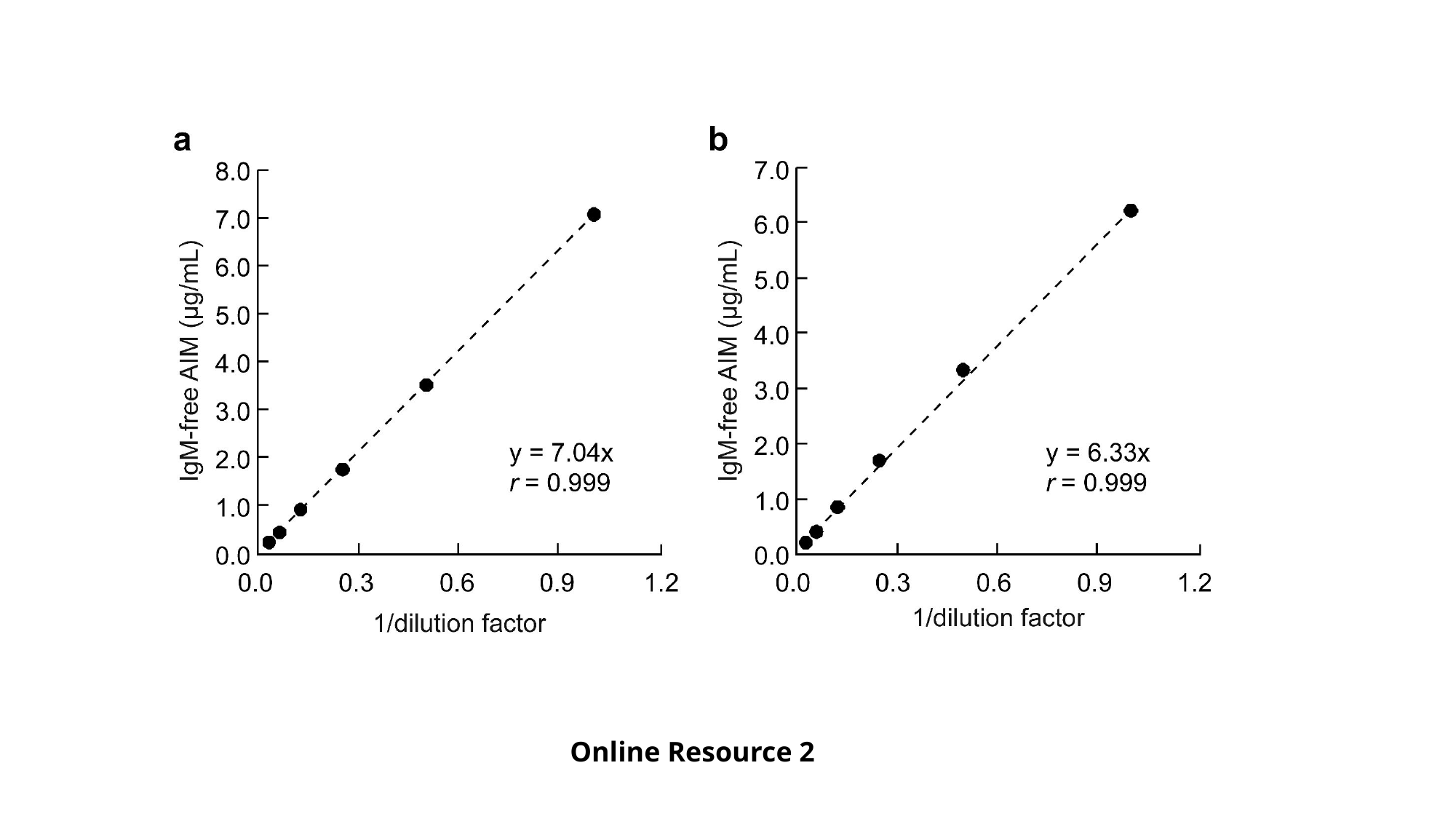

Online Resource 2

## Slide 3
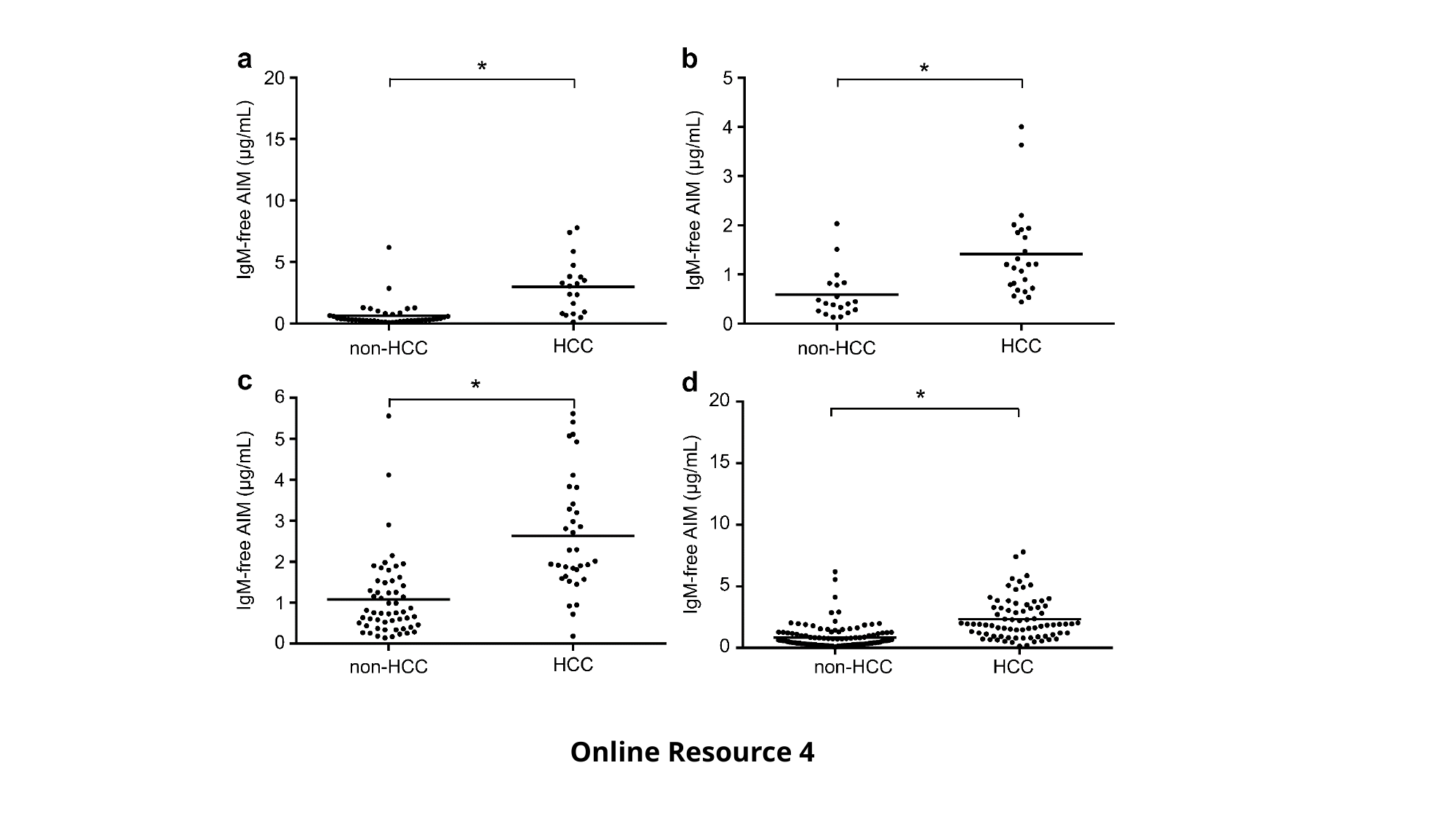

Online Resource 4

## Slide 4
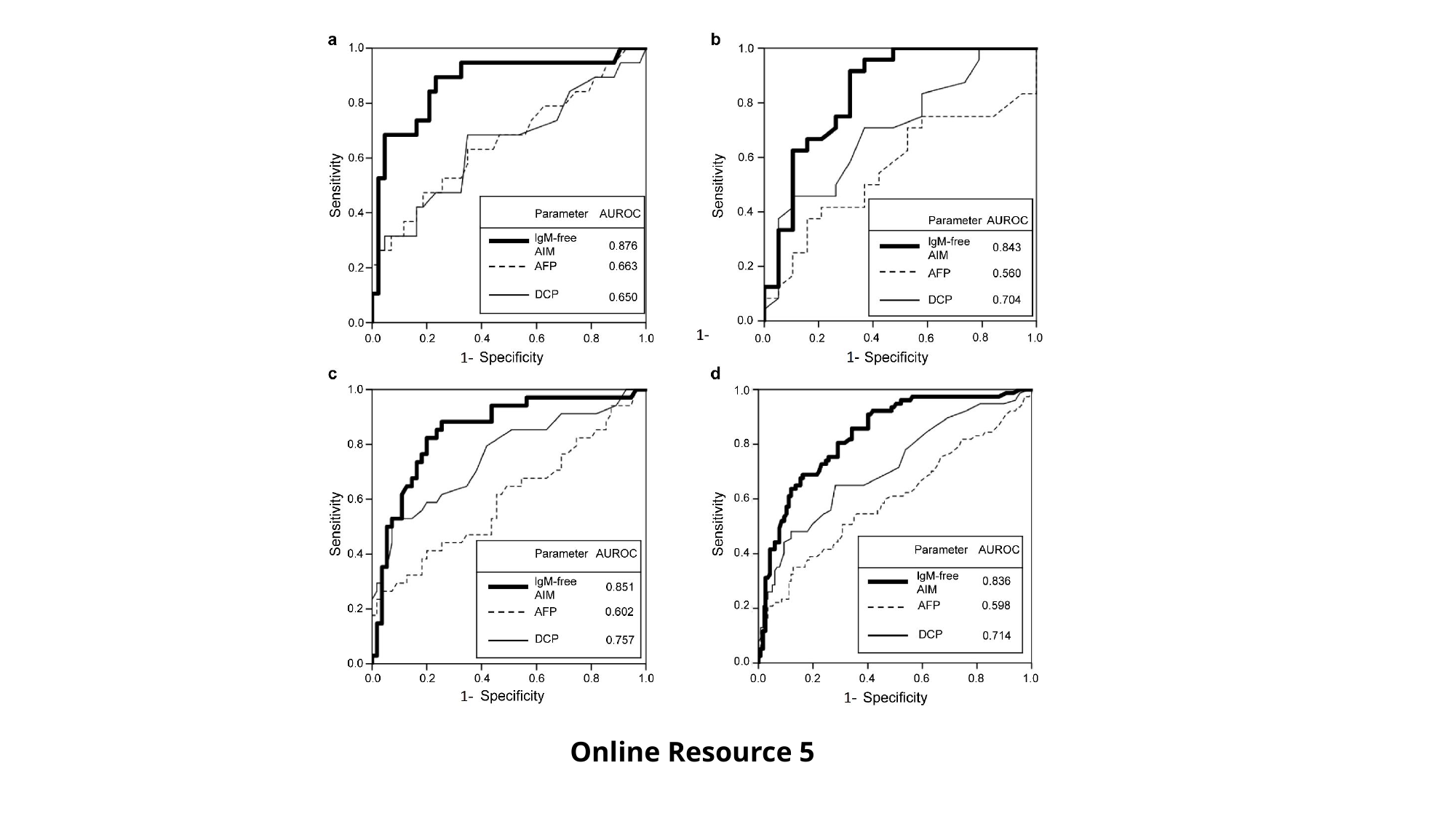

Online Resource 5

## Slide 5
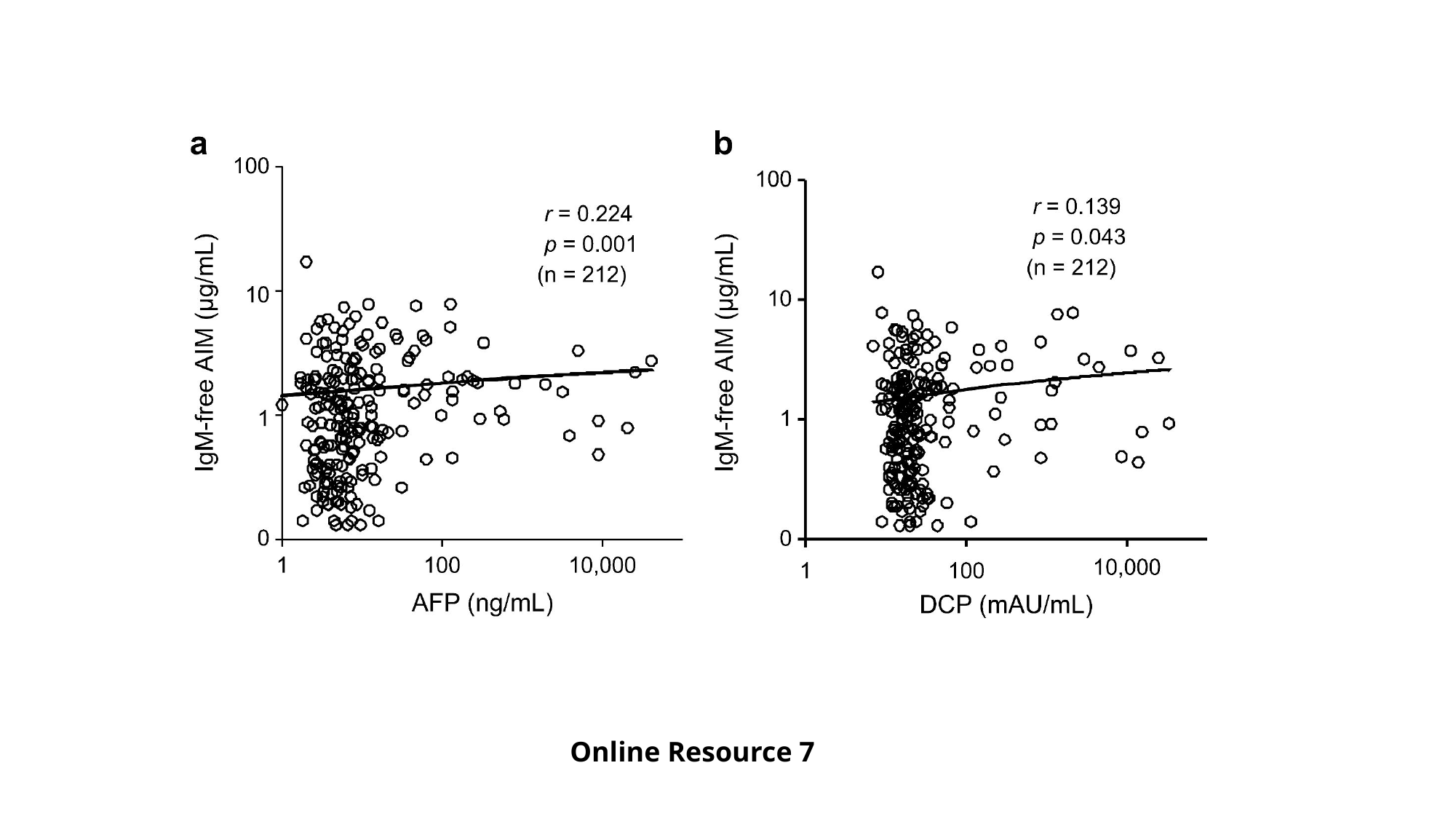

Online Resource 7

## Slide 6
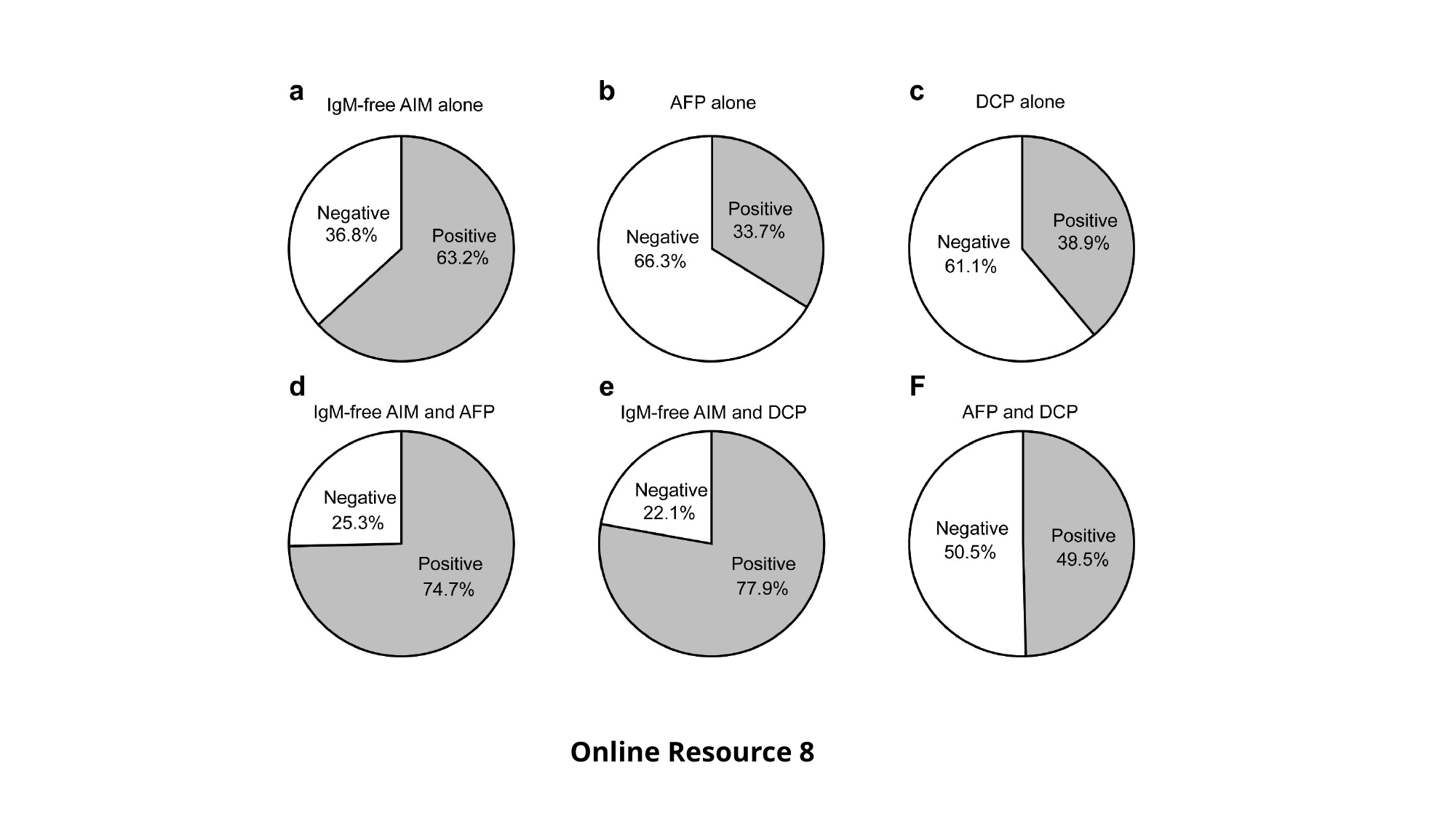

Online Resource 8

## Slide 7
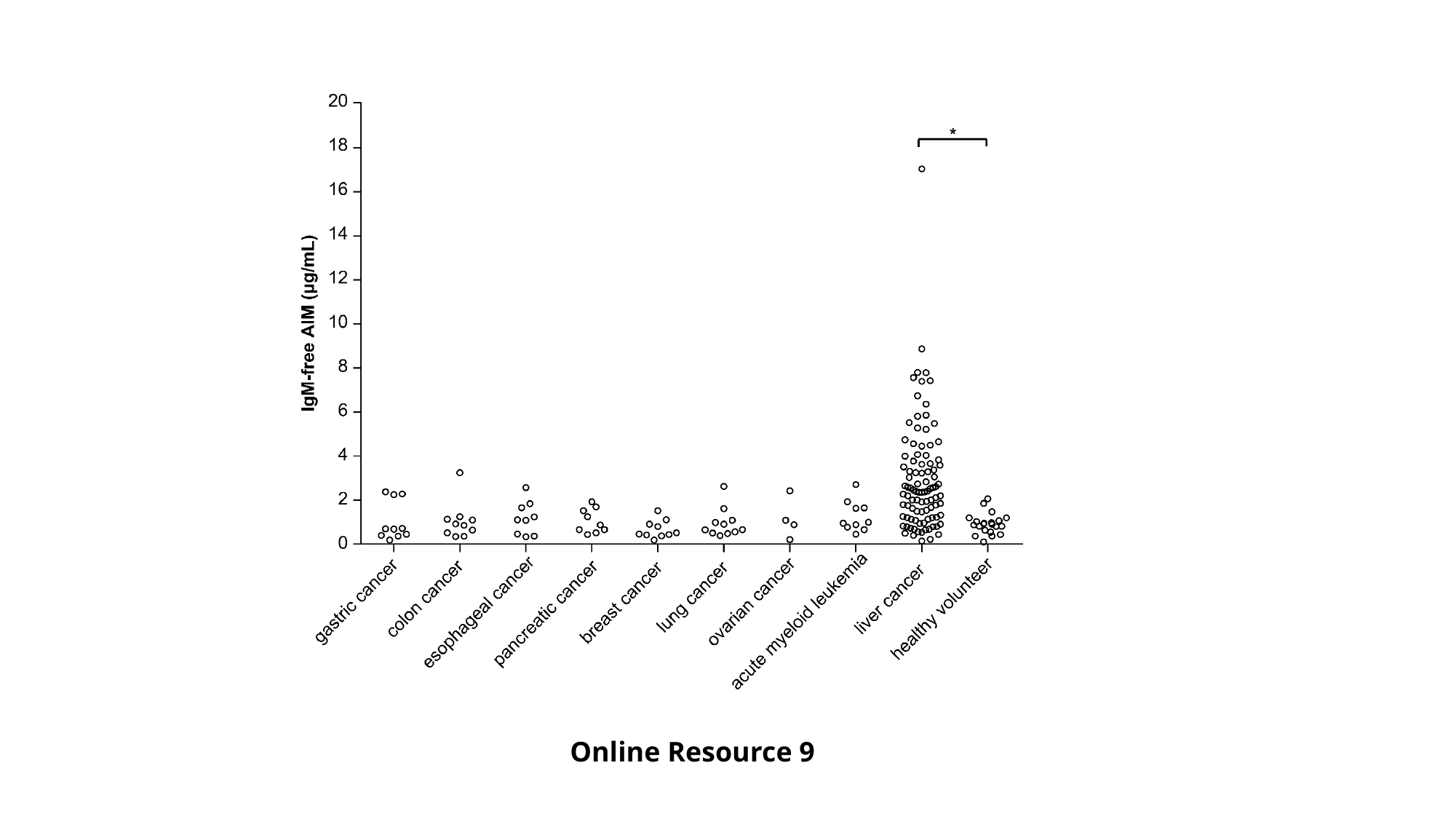

Online Resource 9

## Slide 8
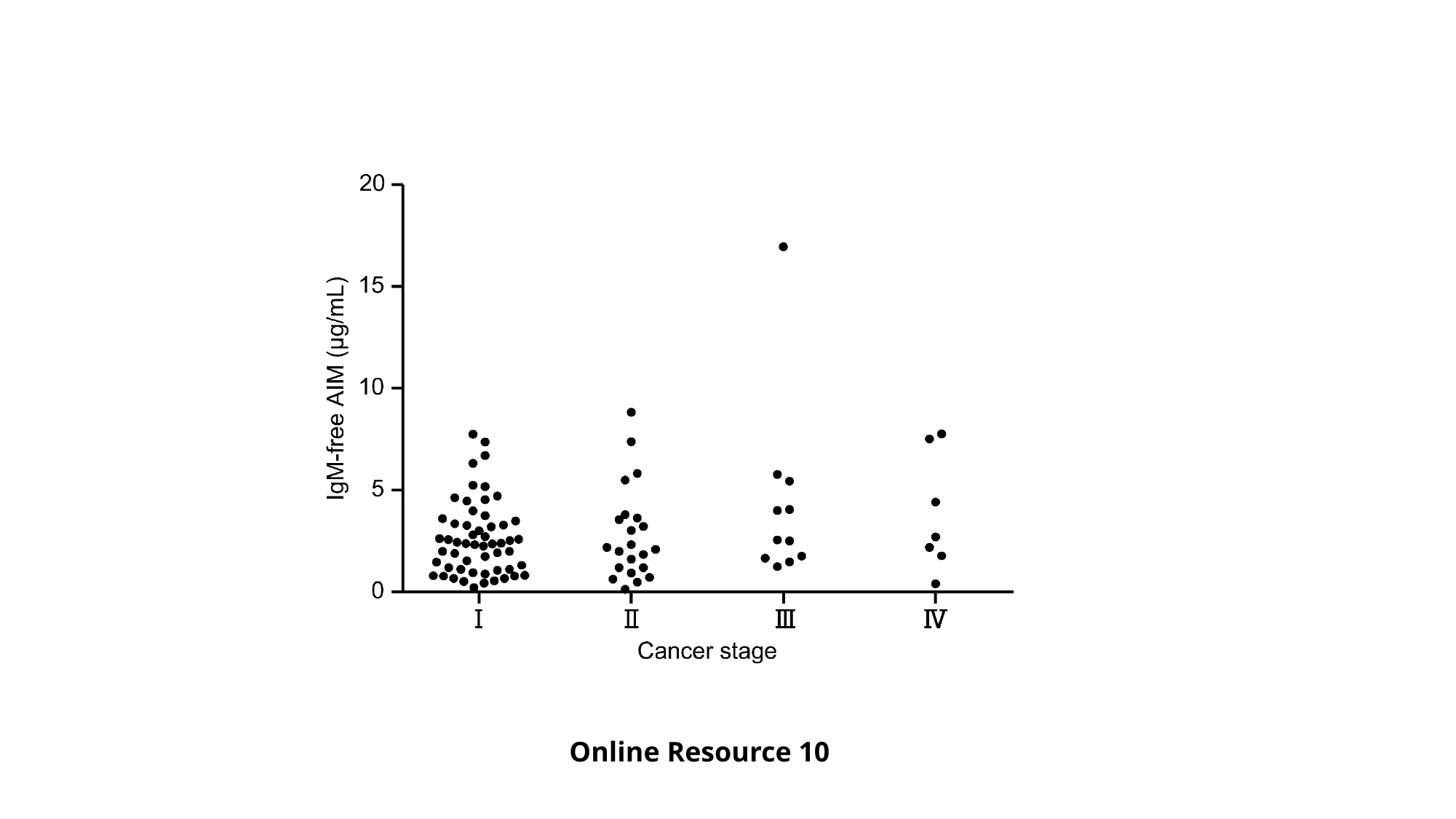

Online Resource 10

## Slide 9
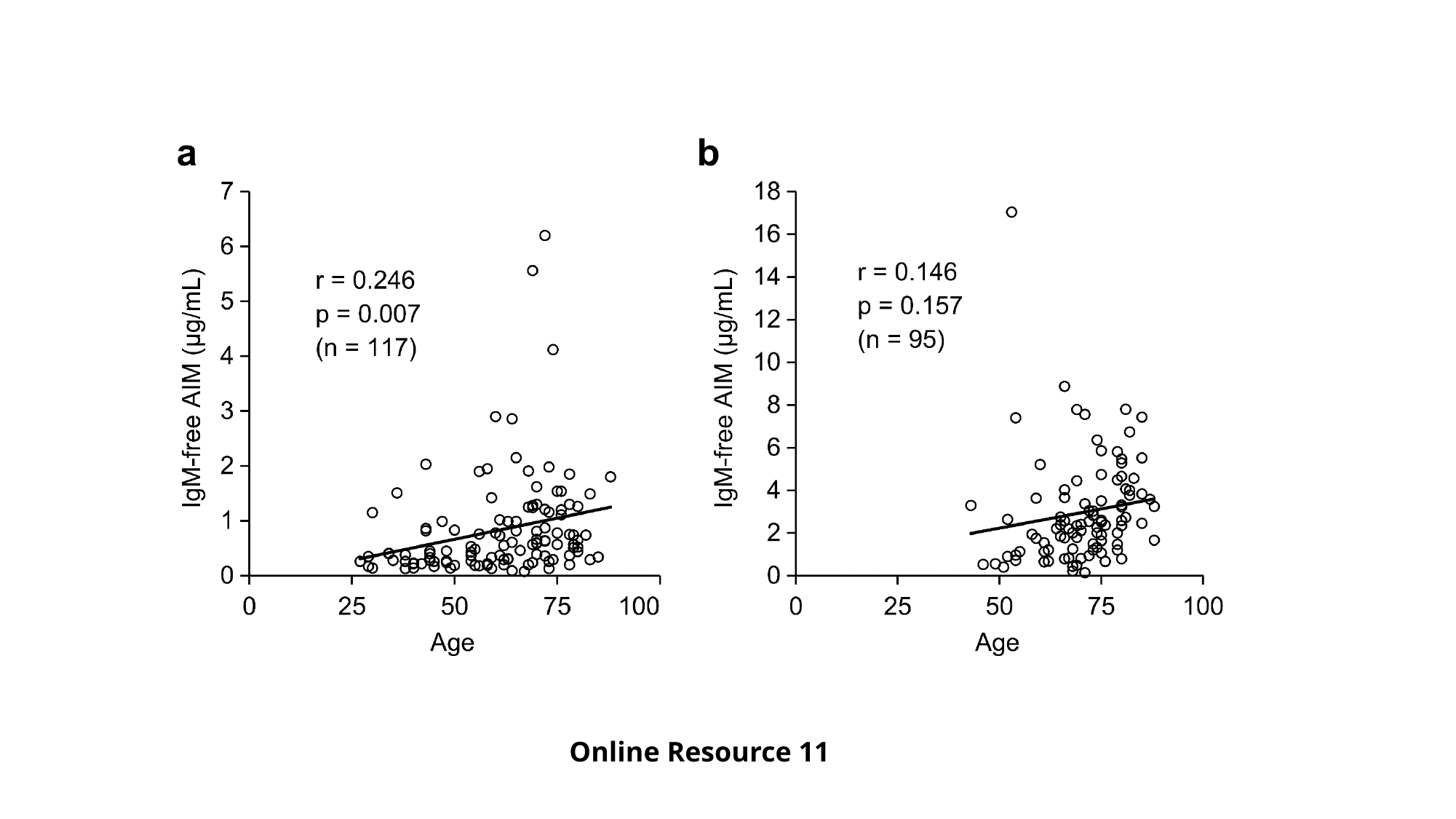

Online Resource 11

## Slide 10
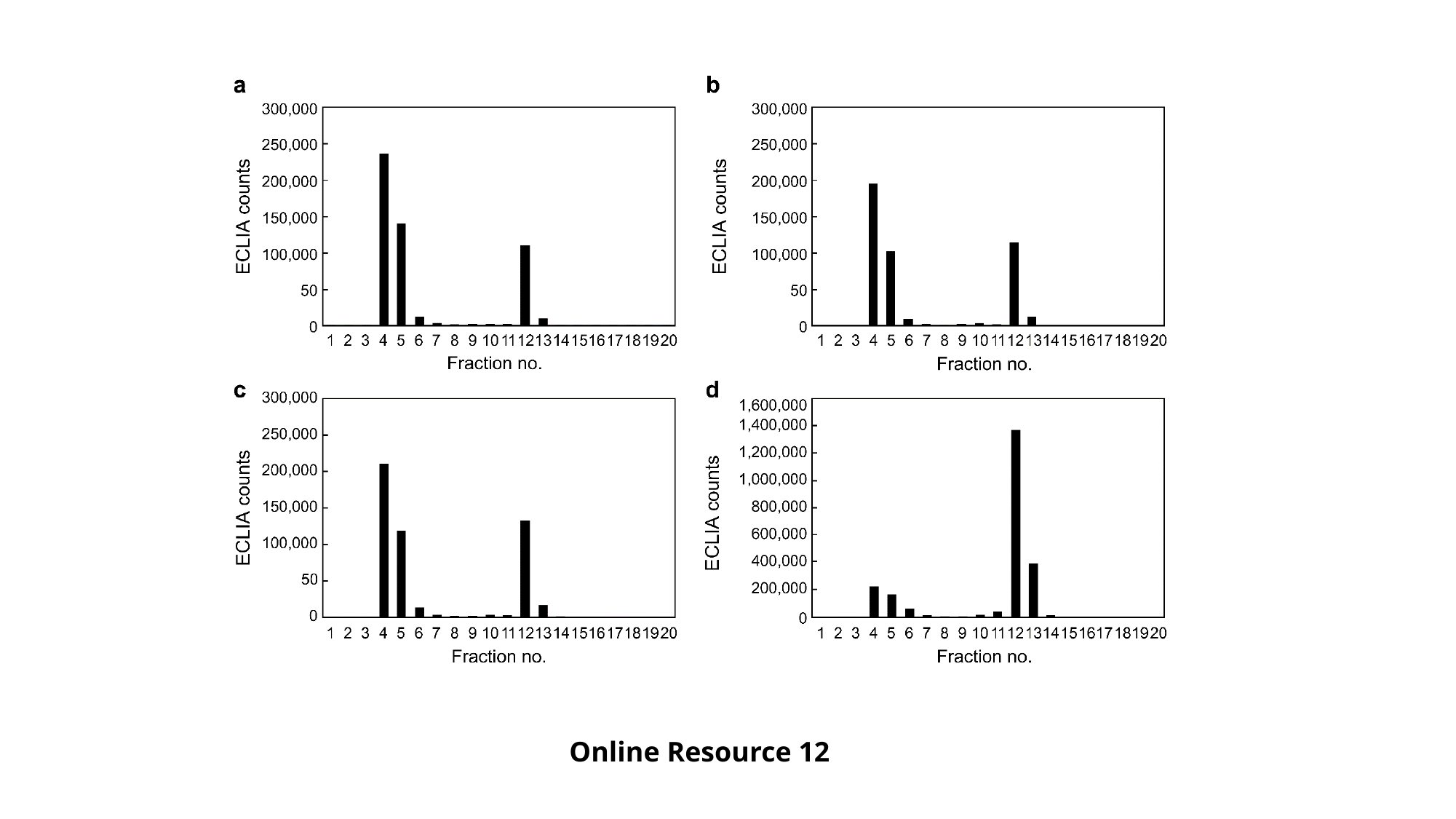

Online Resource 12
