# Supplementary material for: Hepatocellular carcinoma diagnosis using a novel electrochemiluminescence immunoassay targeting serum IgM-free AIM
Source: Clin J Gastroenterol. 2022 Jan 4;15(1):41–51. doi: 10.1007/s12328-021-01567-4 (PMC8858287; doi:10.1007/s12328-021-01567-4)
Supplement: Supplementary file 2 — Supplementary file2 (DOCX 33 kb) [file 12328_2021_1567_MOESM2_ESM.docx]

**Hepatocellular carcinoma diagnosis using a novel electrochemiluminescence immunoassay targeting serum IgM-free AIM**

Tomo Shimizu^1*^, Takashi Sawada^1^, Tomohide Asai^1^, Yuka Kanetsuki^1^,

Jiro Hirota^1^, Michihisa Moriguchi^2^, Tomoaki Nakajima^3^, Toru Miyazaki^4, 5, 6, 7^, and Takeshi Okanoue^8^

^1^ Tsukuba Research Institute Research & Development Division, Sekisui Medical Co., Ltd., Ibaraki 301-0852, Japan

^2^ Department of Molecular Gastroenterology and Hepatology, Graduate School of Medical Science, Kyoto Prefectural University of Medicine, Kyoto 602-8566, Japan

^3^ Department of Hepatology, Sapporo Kosei General Hospital, Hokkaido 060-0033, Japan

^4^ Laboratory of Molecular Biomedicine for Pathogenesis, Center for Disease Biology and Integrative Medicine, Faculty of Medicine, The University of Tokyo, Tokyo 113-0033, Japan

^5^ LEAP, Japan Agency for Medical Research and Development, Tokyo 113-0033, Japan

^6^ Laboratoire d'ImmunoRhumatologie Moléculaire, Plateforme GENOMAX, Institut National de la Santé et de la Recherche Médicale UMR_S 1109, Faculté de Médecine, Fédération Hospitalo-Universitaire OMICARE, Fédération de Médecine Translationnelle de Strasbourg, Laboratory of Excellence TRANSPLANTEX, Université de Strasbourg, Strasbourg, France

^7^ The Institute for AIM Medicine, Tokyo 101-0047, Japan

^8^ Department of Gastroenterology and Hepatology, Saiseikai Suita Hospital, Osaka 564-0013, Japan

***Corresponding author:**

Tomo Shimizu

Tsukuba Research Institute Research & Development Division, Sekisui Medical Co., Ltd., 3-3-1, Koyodai, Ryugasaki, Ibaraki 301-0852, Japan

TEL: +81-297-62-6425

FAX: +81-297-62-8635

E-mail: tomo_shimizu@sekisui.com

**Online Resource 1. Correlation between IgM-free AIM level (µg/mL) via direct ECLIA and ECLIA counts of IgM-free AIM fractions obtained by size-exclusion chromatography.** IgM-free AIM levels in serum by direct ECLIA using anti-AIM antibody No. 12 and No. 11 were significantly correlated with those of the IgM-free AIM fractions obtained by size-exclusion chromatography. Abbreviations: AIM, apoptosis inhibitor of macrophage; ECLIA, electrochemiluminescence immunoassay.

**Online Resource 2. Sample dilution linearity of two serum samples from patients (a) No. 1 and (b) No. 2 with NASH-HCC.** Each serum sample was serially diluted from 1:1 to 1:64 and IgM-free AIM was measured by ECLIA using beads coated with anti-AIM antibody No. 12 and Ru-labeled anti-AIM antibody No. 11. Good dilution linearity was observed for each serum sample. Abbreviations: AIM, apoptosis inhibitor of macrophage; ECLIA, electrochemiluminescence immunoassay; HCC, hepatocellular carcinoma; NASH, non-alcoholic steatohepatitis.

**Online Resource 3. The effect of interfering substances on bilirubin F and C, hemoglobin, chyle, and rheumatoid factor**

| Specimen | Interfering substance spiked | | IgM-free AIM (µg/mL) | | |
| --- | --- | --- | --- | --- | --- |
|  |  |  | Assay 1 | Assay 2 | Average |
| No. 1 | Bilirubin F | 0 mg/dL | 0.57 | 0.61 | 0.59 |
|  | 25 mg/dL | 0.53 | 0.57 | 0.55 |  |
| Bilirubin C | 0 mg/dL | 0.56 | 0.54 | 0.55 |  |
|  | 25 mg/dL | 0.55 | 0.54 | 0.55 |  |
| Hemoglobin | 0 mg/dL | 0.53 | 0.56 | 0.55 |  |
|  | 500 mg/dL | 0.55 | 0.56 | 0.56 |  |
| Chyle | 0 FTU | 0.56 | 0.55 | 0.56 |  |
|  | 3,000 FTU | 0.56 | 0.56 | 0.56 |  |
| Rheumatoid factor | 0 IU/mL | 0.61 | 0.63 | 0.62 |  |
|  |  | 500 IU/mL | 0.64 | 0.63 | 0.64 |
| No. 2 | Bilirubin F | 0 mg/dL | 2.19 | 2.13 | 2.16 |
|  | 25 mg/dL | 2.15 | 2.24 | 2.20 |  |
| Bilirubin C | 0 mg/dL | 2.10 | 2.15 | 2.13 |  |
|  | 25 mg/dL | 2.27 | 2.13 | 2.20 |  |
| Hemoglobin | 0 mg/dL | 2.20 | 2.18 | 2.19 |  |
|  | 500 mg/dL | 2.24 | 2.28 | 2.26 |  |
| Chyle | 0 FTU | 2.18 | 2.21 | 2.20 |  |
|  | 3,000 FTU | 2.20 | 2.13 | 2.17 |  |
| Rheumatoid factor | 0 IU/mL | 2.32 | 2.27 | 2.30 |  |
|  |  | 500 IU/mL | 2.37 | 2.21 | 2.29 |

AIM, apoptosis inhibitor of macrophage

**Online Resource 4. Distribution mapping of serum IgM-free AIM levels in non-HCC (hepatitis and cirrhosis) and HCC patient groups for cancer stage 1 and 2.** The results shown in (a), (b), (c), and (d) are for cancer stage 1 and 2 of NASH-HCC, HBV-HCC, HCV-HCC, and all the patients together, respectively. The serum IgM-free AIM level was significantly higher in the HCC patient group than in the non-HCC patient group (*p* < 0.001) regardless of the pathogenesis. **p* < 0.001 (Mann–Whitney U-test). Abbreviations: AIM, apoptosis inhibitor of macrophage; HBV, hepatitis B virus; HCC, hepatocellular carcinoma; HCV, hepatitis C virus; NASH, non-alcoholic steatohepatitis.

**Online Resource 5. ROC analyses of IgM-free AIM, AFP, and DCP for cancer stage 1 and 2.** The sensitivity and specificity of the markers were determined to distinguish between the patients with HCC and those without HCC. (a), (b), (c), and (d) include cancer stage 1 and 2 of NASH-HCC, HBV-HCC, HCV-HCC, and all the patients, respectively. IgM-free AIM showed the highest AUROC for each disease group and all the patients. Abbreviations: AFP, alpha-fetoprotein; AIM, apoptosis inhibitor of macrophage; DCP, des-γ-carboxy prothrombin; HBV, hepatitis B virus; HCC, hepatocellular carcinoma; HCV, hepatitis C virus; NASH, non-alcoholic steatohepatitis; ROC, receiver operating characteristic; AUROC, area under the receiver operating characteristic.

**Online Resource 6. Comparison of the sensitivity, specificity, and accuracy of IgM-free AIM, AFP, and DCP for identifying patients with HCC of cancer stage 1 and 2**

|  | **Marker** | **Sensitivity** | **Specificity** | **Accuracy** |
| --- | --- | --- | --- | --- |
| Comparison of diagnostic performance for NASH-HCC | | | | |
| **Stage 1 and 2** | IgM-free AIM | 68.4% | 95.3% | 87.1% |
|  | AFP | 5.3% | 100% | 71.0% |
|  | DCP | 31.6% | 90.7% | 72.6% |
| Comparison of diagnostic performance for HBV-HCC | | | | |
| **Stage 1 and 2** | IgM-free AIM | 33.3% | 94.7% | 60.5% |
|  | AFP | 25.0% | 84.2% | 51.2% |
|  | DCP | 29.2% | 94.7% | 58.1% |
| Comparison of diagnostic performance for HCV-HCC | | | | |
| **Stage 1 and 2** | IgM-free AIM | 88.2% | 80.0% | 83.1% |
|  | AFP | 32.4% | 87.3% | 66.3% |
|  | DCP | 32.4% | 96.4% | 71.9% |
| Comparison of diagnostic performance for all HCC patients | | | | |
| **Stage 1 and 2** | IgM-free AIM | 66.2% | 88.0% | 79.4% |
|  | AFP | 23.4% | 91.5% | 64.4% |
|  | DCP | 31.2% | 94.0% | 69.1% |

The cut-off values of IgM-free AIM, AFP, and DCP were set at 1.6 µg/mL, 20 ng/mL, and 40 mAU/mL, respectively.

Abbreviations: AFP, alpha-fetoprotein; AIM, apoptosis inhibitor of macrophage; DCP, des-γ-carboxy prothrombin; HBV, hepatitis B virus; HCC, hepatocellular carcinoma; HCV, hepatitis C virus; NASH, non-alcoholic steatohepatitis

**Online Resource 7. Correlation between IgM-free AIM and AFP or DCP.** IgM-free AIM did not show a definite correlation with (a) AFP or (b) DCP. Abbreviations: AFP, alpha-fetoprotein; AIM, apoptosis inhibitor of macrophage; DCP, des-γ-carboxy prothrombin.

**Online Resource 8. Positivity rate of the combination assay of IgM-free AIM with AFP and/or DCP.** (a) IgM-free AIM alone; (b) AFP alone; (c) DCP alone; (d) combination of IgM-free AIM and AFP; (e) combination of IgM-free AIM and DCP; and (f) combination of AFP and DCP. The positivity rate of IgM-free AIM in combination with AFP or DCP was higher than that of the combination of AFP and DCP. Abbreviations: AFP, alpha-fetoprotein; AIM, apoptosis inhibitor of macrophage; DCP, des-γ-carboxy prothrombin.

**Online Resource 9. Distribution mapping of serum IgM-free AIM level for each cancer type and healthy volunteer.** IgM-free AIM was significantly high only for liver cancer (*p* < 0.001) and was not elevated in other types of cancer when compared to normal volunteers. **p* < 0.001 (Mann–Whitney U-test). Abbreviations: AIM, apoptosis inhibitor of macrophage.

**Online Resource 10. Distribution mapping of serum IgM-free AIM level at each cancer stage.** There was no difference in the IgM-free AIM level among patients at each cancer stage. Abbreviations: AIM, apoptosis inhibitor of macrophage.

**Online Resource 11. Correlation between IgM-free AIM and age.** IgM-free AIM did not show a definite correlation with the age of patients regardless of whether they were in the (a) non-HCC or (b) HCC group. Abbreviations: AIM, apoptosis inhibitor of macrophage; HCC, hepatocellular carcinoma.

**Online Resource 12. ECLIA counts of size-exclusion chromatography fractionated serum samples from healthy volunteers and from patients with NAFL, NASH, and NASH-HCC.** ECLIA counts of fractionated serum samples from (a) healthy volunteers and patients with (b) NAFL, (c) NASH, and (d) NASH-HCC. ECLIA using beads coated with anti-AIM antibody No. 11 and Ru-labeled anti-AIM antibody No. 8 was used to measure both IgM-free and IgM-bound AIM. The peaks of fractions No. 4–6 (IgM-bound AIM) from (a) healthy volunteers and from patients with (b) NAFL, and (c) NASH were higher than those of fractions No. 12–13 (IgM-free AIM), whereas the peaks of fractions No. 12–13 (IgM-free AIM) from the serum of patients with (d) NASH-HCC were higher than those of fractions No. 4–6. Abbreviations: AIM, apoptosis inhibitor of macrophage; ECLIA, electrochemiluminescence immunoassay; HCC, hepatocellular carcinoma; NAFL, non-alcoholic fatty liver; NASH, non-alcoholic steatohepatitis.
